# Laboratory spiking process of soil with various uranium and other heavy metals

**DOI:** 10.1016/j.mex.2019.03.026

**Published:** 2019-04-03

**Authors:** Liangmei Chen, Steven L. Larson, John H. Ballard, Youhua Ma, Qinku Zhang, Jiangxia Li, Linchun Wu, Zikri Arslan, Fengxiang X. Han

**Affiliations:** a Department of Chemistry and Biochemistry, Jackson State University, 1400 J. R. Lynch Street, P.O. Box 17910, Jackson, MS, 39217, USA; b College of Resource and Environment, Anhui Agricultural University, Hefei, Anhui, China; c U.S. Army Engineer Research and Development Center, 3909 Halls Ferry Rd., Vicksburg, MS, 39180-6199, USA

**Keywords:** Spiking soil with metal salt powder, Spiking soil, Total uranium, Contamination, Heavy metals

## Abstract

Laboratory studies using metal spiked soils are challenging due to soil heterogeneity. This work provides an easy, quick, precise, and accurate technique for the preparation of spiked soils for laboratory research. The process described spiking soil with various uranium species and other heavy metals for laboratory scale pilot experiments under various biogeochemical conditions. The procedure involves grinding both dry soil and metal chemicals into the fine powder. The spiked soil mixture was further homogenized through a modified splitting and combining of the sample by diagonal flipping using plastic sheeting. Comparison of measured concentrations with theoretical values were obtained with <20% precision and accuracy. However, tradition spiking method with metal solution often yielded high heterogeneous spiked soils due to strong metal adsorption in soils. Re-drying and re-grinding of soils were required following the spiking in order to homogenize treated soils, generating inhalable particulates. Thus appropriate personal protective equipment and practices are required for the safety concern. The present method with metal salt powder proved a safe, useful, quick, accurate and precise, and homogenized soil spiking method.

• ability to prepare spiked soil with multiple elements
• prepared soil at any level of loading
• the spiked soil was homogenous for controlled studies

## Graphical abstract

**Figure:**
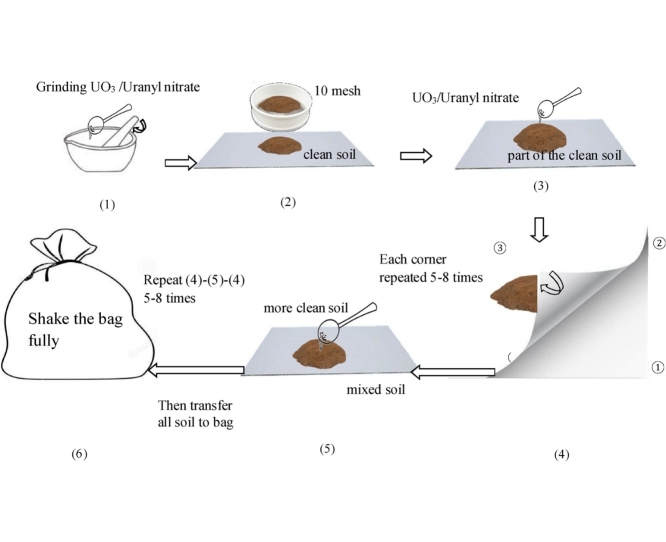

**Specifications Table**

**Table:** 

Subject Area:	Environmental Science
More specific subject area:	Heavy metal pollution and control
Method name:	Spiking soil with metal salt powder
Name and reference of original method:	F.X. Han, A. Banin. Long-term transformations and redistribution of potentially toxic heavy metals in arid-zone soils incubated: I. Under saturated conditions. Water, Air, and Soil Pollution, 95 (1997), PP. 399–423 [4].
	F.X. Han, A. Banin. Long-term transformation and redistribution of potentially toxic heavy metals in arid-zone soils: II. Incubation at the field capacity moisture content. Water, Air, and Soil Pollution, 114 (1999), PP. 221–250 [5].
Resource availability:	N/A

## Method details

### Overview

Soil pollution poses a significant risk to ecosystems and human health. The remediation of contaminated soil is a global challenge for sustainability of ecosystems and social development. Generally speaking, remediation of contaminated soils requires a series of experimental controlled studies and environmental assessment of its efficiency [1]. The key to the success of such experiments is that the soil used in replicates and operational conditions is uniform to the extent so that statistically significant comparisons of the processes can be made. Sometimes, potential collection sites for obtaining contaminated field samples are restricted due to access, regulatory, or license factors. Other factors that necessitate the production of manufactured samples for laboratory studies is the heterogeneous nature of field samples. Use of field soils that are contaminated with multiple forms of metallic contaminants such as zero valent metals, metal oxides, and metal salts or multiple metal species with regards to oxidative or coordinative variability is challenging. These soils often require extensive particle size reduction and other homogenization processes in order to produce representative subsamples. Use of these processing steps often results in subsamples with homogeneous metal concentrations but biogeochemical properties that have changed significantly when compared to the original field sample [2]. Many of these issues can be resolved through the production of soil subsamples for remediation studies that represent specific field conditions and are representative of one another. This requires laboratory/green-house controlled study through spiking metal pollutants into soils, i.e., to mix exogenous pollutants into the soil to prepare polluted soils with desired metal compositions, forms and species, and specific loading levels [3,4]. The main advantages of the soil manufacture for laboratory remediation studies with various uranium species are superior convenience and the ability to prepare homogenous representative contaminated soil with multiple elements to simulate various scenarios safely.

### Procedures

1 Air dry the characterized soil selected to meet study requirements.
2 Grind the dry soil using wood plate and grinding rod
3 Pass the ground soil over 2 mm sieve
4 Measure the mass of metal species (UO_3_, Uranyl nitrate hexa-hydrate) based on the desired loading level and the desired weight of the soil
5 Completely grind the metal compounds into the final powder (solid UO_3_ and Uranyl nitrate hexa-hydrate) with agate mortar
6 Grind about 25–50 g soil into the fine powder with agate mortar (in general 10–20 times metal salt mass)
7 Mix well the ground metal compounds with the fine ground of soil inside the mortar with rods
8 Place a mixture in the center of a clean plastic sheet (2 m*2 m)
9 Diagonally flip the corners of the plastic paper to the center, repeat 5–8 times.
10 Gradually and progressively add more bulk soils (about 10–20 times the mixture at each addition till all clean soil was added) into the homogenized mixture and continue with the diagonal flipping the corners of the plastic paper for 5–8 times
11 Pour the mixed homogenized treated soil into a plastic bag, continuously invert the mixed soil in the bag, repeat 5–8 times. Divide the mixed treated contaminated soils into evenly subsampled container in order to produce the desired number of representative subsamples for laboratory study replicates and conditions for controlled experiments [3,4].
12 All steps should be carried out in laboratory hoods to avoid harm to workers. Precautions should be employed with proper procedures and protective equipment when dealing with heavy metals and U compounds. Hoods, dust masks, double gloves, tyvek suits, rad-detector/survey tools are required for U works. Proper rad decontamination procedures should be followed, such as discarding rad contaminated sheets, gloves, masks, Tyvek suits in the separate designated rad container for proper handling. Finally all personals should be checked for potential rad contamination with proper rad survey meters to ensure the personnel safety.

### Final remarks

The present spiking method with metal powder provides a safe, easy, quick, and accurate, homogenized spiking process of soil with various uranium spices and other heavy metals. The spiked process showed reasonable precision among replicates and good recoveries compared to theoretical values. The procedure mainly involved grinding dry soil through 2 mm sieve and grinding metal chemicals into the fine powder in the agate mortar. Metal compounds were slowly and progressively mixed with ground dry soil (as 10–20 times the mass of metal compounds) and the mixture were continued to expand to cover all the soil. Finally the spiked soil mixture was homogenized through diagonal flipping soil on the plastic sheet for 3–5 times. Compared to other traditional spiking method such as liquid addition of metal solutions into the soil, the current spiking method easily generated more homogenous contaminated soils with desired metal loading levels of multiple metals at specific ratios. The liquid spiking method was hard to mix the soil completely due to strong metal adsorption and localization of metals in soils, resulting in strong heterogeneity in soils inside the containers. Re-drying the treated soil and re-grinding the contaminated soil were required in order to homogenize the treated soils, which often generated much dusts, resulting in safety concerns to workers. Thus the present spiking method with grinding metal powder proved a safe, useful, quick, and homogenized soil spiking method.

#### Measuring procedures of the total uranium and other heavy metal concentration in the mixed soil samples

1 Weigh 1.0 g soil sample into digesting tube with duplicates (50 ml)
2 Digest the soil with twenty-five ml of 4 M HNO_3_ in a water-bath at 80 °C for 16 h [3,4]
3 The supernatant was decanted and filtered through a 0.45-μm filter
4 Dilute the digested soil solution
5 Measure the metal concentrations in the diluted solution with ICP-MS

#### Verifying the validity of spiking process of soil with various uranium spices

**Fig. 1:**
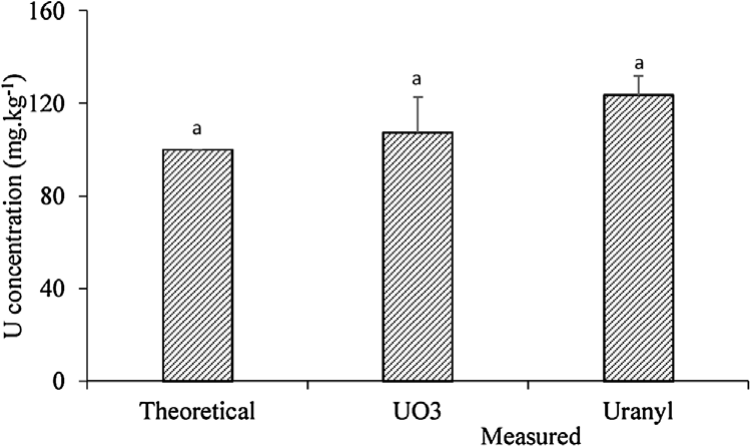
The comparison of the U concentration between measured values of spiking soils and the theoretical data. The same lower case indicates no significant difference at p = 0.05 level between measured values and theoretical data of the spiking soils.

**Fig. 2:**
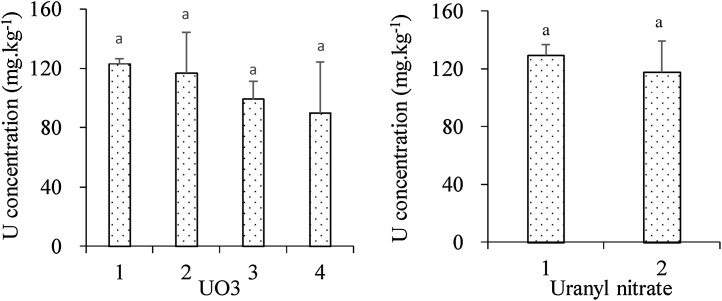
Uranium concentration in the soil spiked with uranium in different containers. The same lowercase showed no significant difference in U concentrations in the soils between different containers.

Fig. 1 illustrated that spiking method with metal salt powder generated a reasonable spiked contaminated soils with U species. The measured values were consistent with theoretical results. As required in order to meet study needs, no significant difference (p < 0.05 probability) in the uranium concentration in the soils between different uranium spices treatments was observed. Fig. 2 showed that there was also no significant difference (p < 0.05 probability) in the uranium concentration among various replicates in the same treated soil where two subsamples were taken to analyze the total metal concentration from each replicate (container) even though spatial variation did exist among containers (replicates). The coefficient of variation of UO_3_ and uranyl nitrate were 14.3% and 6.7%, respectively. This indicates the reasonable precision and repeatability of the method in spiking contaminated soils with various species of U required for phytoremediation greenhouse studies under controlled conditions.

**Fig. 3:**
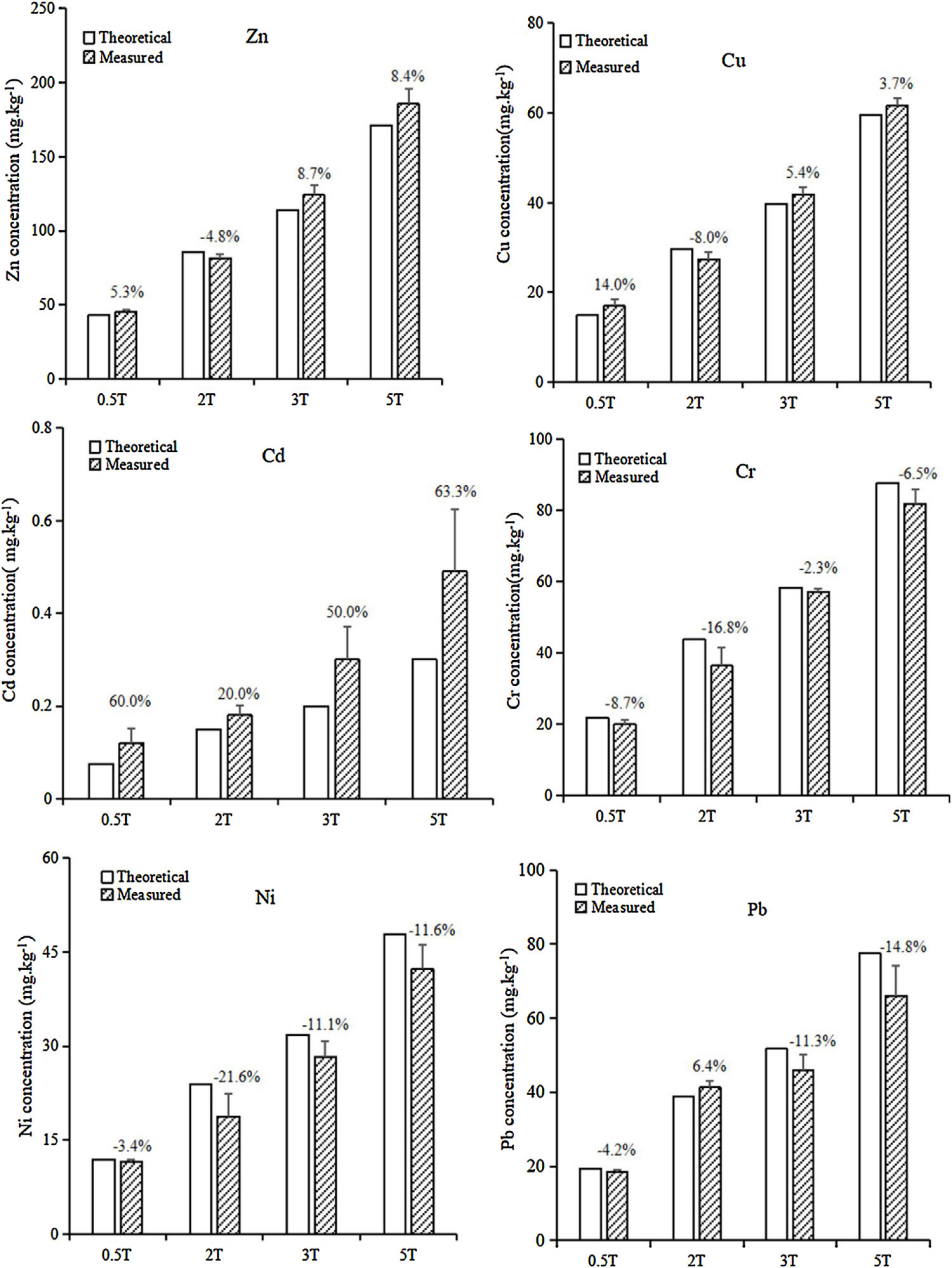
Comparison of the measured concentrations and theoretical values of Cu, Cr, Pb, Cd, Ni, Pb and Zn in metal salt spiked arid zone soils (the data were from [[4], [5], [6]]). The percentage was the CV% between the measured and theoretical values of heavy metals in spiked soils. T denotes the total metal concentrations in the original untreated soil.

The same spiking method was used with metal salt powders to treat arid zone soils with Zn, Cu, Cd, Cr, Pb and Ni [[4], [5], [6]]. Experimental data indicated for all treatments of six metals except Cd, the CV% between the theoretical values and measured values were below 20% (Fig. 3). The exception was Cd treatments with high CV% since added Cd was at low loading levels. This could be overcome with additional mixing steps if increased sample homogeneity was required for a given study. The use of the production method in prepared heavy metal contaminated soil has been shown to be effective [[4], [5], [6]]. Six heavy metals were mixed together first with 4 of loading levels. There was no significant difference (p < 0.05 probability) between the spiked results of Ni, Cd, Cr and theoretical data. The standard deviation of the three metal elements were all less than 5.5%. Therefore, the proposed spiking methodology may be efficiently applied for spiking soils with various contaminants.

Compared to other common spiking method such as addition of metal solutions into the soil [7,8], the current spiking method with metal powder produced representative subsamples with biogeochemical properties required for experimentally determining the effectiveness of specific remediation techniques. For traditional spiking method with metal containing solution, it is hard to mix the soil with metals completely, resulting in high heterogeneity among micro-positons of soils inside the containers due to strong adsorption and strong localization of added heavy metals in the soil. Also the traditional methods often requires re-drying the treated soil and re-grinding the contaminated soil in order to homogenize the treated soils. Grinding treated polluted soils often caused dusts, resulting in safety concerns to workers.

Using dissolved metals can produce homogeneous samples but soil properties are changed in the process such as soil pH, soil microbial community, dissolution/precipitation events, etc. The current spiking method with grinding metal salt powder mimicked “solid solution” approach, resulting in manufactured soils that are more useful for laboratory/greenhouse research and that are superior to liquid addition and post evaporation homogenization. This approach can also be used to introduce mixed metal forms such as zero valent oxides and salts at known ratios for consistent initial contaminant conditions across replicates and treatments.
